# Multi-year aboveground data of minirhizotron facilities in Selhausen

**DOI:** 10.1038/s41597-024-03535-2

**Published:** 2024-06-22

**Authors:** Thuy Huu Nguyen, Gina Lopez, Sabine J. Seidel, Lena Lärm, Felix Maximilian Bauer, Anja Klotzsche, Andrea Schnepf, Thomas Gaiser, Hubert Hüging, Frank Ewert

**Affiliations:** 1 University of Bonn, Institute of Crop Science and Resource Conservation (INRES), Katzenburgweg 5, 53115 Bonn, Germany; 2 Agrosphere (IBG-3), Institute of Bio- and Geosciences, Forschungszentrum Jülich GmbH, 52428 Jülich, Germany; 3 Leibniz Centre for Agricultural Landscape Research (ZALF), Institute of Landscape Systems Analysis, Eberswalder Strasse 84, 15374 Muencheberg, Germany

**Keywords:** Hydrology, Drought, Agriculture

## Abstract

Improved understanding of crops’ response to soil water stress is important to advance soil-plant system models and to support crop breeding, crop and varietal selection, and management decisions to minimize negative impacts. Studies on eco-physiological crop characteristics from leaf to canopy for different soil water conditions and crops are often carried out at controlled conditions. In-field measurements under realistic field conditions and data of plant water potential, its links with CO_2_ and H_2_O gas fluxes, and crop growth processes are rare. Here, we presented a comprehensive data set collected from leaf to canopy using sophisticated and comprehensive sensing techniques (leaf chlorophyll, stomatal conductance and photosynthesis, canopy CO_2_ exchange, sap flow, and canopy temperature) including detailed crop growth characteristics based on destructive methods (crop height, leaf area index, aboveground biomass, and yield). Data were acquired under field conditions with contrasting soil types, water treatments, and different cultivars of wheat and maize. The data from 2016 up to now will be made available for studying soil/water-plant relations and improving soil-plant-atmospheric continuum models.

## Background & Summary

World population predicted to reach 9.8 billion people by 2050 with an expectation of substantial increasing food demand^1^. Due to climate change, water scarcity and drought have frequently occurred in many world regions which have negative impacts to agricultural production and threaten food security^2^. Soil water is one of the most important environmental variables that affects plant water-related processes, gas fluxes (photosynthetic and transpiration), and crop yield^1^. The exchange processes of CO_2_ and H_2_O between crops and their surrounding atmosphere is important for the biomass production and yield. The gas exchange processes also influences the soil-vegetation-atmosphere continuum (SVAC) (e.g., gas and heat flux partitioning) which affects atmospheric and climatic conditions at larger scales^2,3^. In this context, quantification of photosynthesis and transpiration at both leaf and canopy scales, crop growth, and plant hydraulic information is essential to understand water flow and carbon exchange capacity in the SVAC^4^. A collection of such data will also support efficient irrigation, suitable crop practices, and breeding procedures for drought resistance under different climate and soil water conditions^5^. The data will facilitate improvements and prediction skills of mechanistic crop models^6^ and land surface model schemes^7,8^ through representing eco-physiological characteristics of crops^9,10^.

Data collection on plant responses to drought stress/and or soil water variability is often carried out in controlled experiments^11–14^. Extrapolating data from the controlled conditions (e.g. pot and greenhouse) to the field conditions requires caution, because there is difference in evaporative demand^14^ or soil depths and substrates^15^ between the field and the controlled trials. Understanding and modeling the whole SVAC involve different and complex processes from soil to root to shoot, and atmosphere. Root-shoot communication^16^ and interactions of hydraulic and/or non-hydraulic signals^17^ play important roles in regulating stomatal functions and gas flux exchange. Also, from modeling point of view, stomatal conductance cannot be modelled in isolation, but must be fully coupled with models of photosynthesis/respiration and the transport of water from soil, through roots, stems and leaves to the atmosphere. There are positive feedbacks between the stomatal function, aboveground biomass, the root length, the total root system hydraulic conductance, and finally plant hydraulic conductance^18,19^. In summary, this emphasizes necessities in acquiring both below and above-ground data under the field conditions in the SVAC studies.

Leaf water potential, photosynthesis, and transpiration data at both leaf and canopy together with crop growth parameters allow to disentangle the short-term responses (stomatal behaviors) in maize and wheat in regulating plant transpiration and photosynthesis across soil types, soil water availability, and growing seasons^20–23^. Also, such data is crucial for investigating the different traits (e.g. stomatal regulations and leaf area change) and their relative importance and contribution to photosynthesis and transpiration, crop water use efficiency, and biomass production^20,24,25^. Furthermore, the data of leaf water potential and continuously measured transpiration is strongly necessary to understand the water potential gradients and underlying relationships (e.g. plant hydraulic conductance) across the SVAC^26,27^. Leaf water potential, stomatal conductance, transpiration, photosynthesis, and crop growth data (leaf area and biomass) together with the below-ground data (root length density and soil water potential over different soil depths) are very crucial data for parameterizing crop models^18,19^, hydrological models^28^, and land surface models^8,29^ towards improvements of CO_2_ and H_2_O fluxes simulations. To test and validate crop models or vegetation modeling subroutines in the land surface models, an additional information on site conditions (vegetation composition, soil texture, weather, and agricultural management) must be required. Thus, the detailed information on site conditions and agronomic practices is indispensable. The dynamics of biomass, leaf area, and final yield are necessary for field calibration and validation of crop models under different soil types and different growing seasons. Also, the continuous transpiration data measured by sap flow sensors and crop information (leaf area and crop height) are the key data to monitor crop water demand^30,31^ and/or design irrigation system^32^ across growing seasons and soil conditions. Recent developments of UAV and remotely sensed techniques could provide various above-ground crop growth parameters^4,33^. However, an extensive validation of UAV images and processing approach is still required to improve the accuracy to capture plot-to-plot variability within the field and different growing seasons^34,35^. Such crop growth, gas fluxes, and canopy temperature data are useful ground-truth references to validate remote sensing application^35,36^.

Despite the clear relevance and needs for many ecosystem processes, such data on plant water potential processes and links between eco-physiological processes and water potential gradient are generally sparse^4^. Comprehensive measurements from leaf and canopy (the above-ground data) which are aggregated into an accessible database (with the below-ground data), to the best of our knowledge, are rare. The lack of data limits our conceptual understanding of biophysical responses to moisture stress and injects large uncertainty into hydrological and land-surface models. As the detailed publication of the below-ground data in Lärm *et al*.^37^, here we present a comprehensive above-ground data set that was collected under field conditions with two soil types, different water treatments, crop species (wheat and maize), and cultivar mixtures in 2016, 2017, 2018, 2020, and 2021. Data for 2022 and the incoming years will be added up in the future. Data collection covers the detailed measurements from leaf to canopy with some variation between project periods. Leaf photosynthesis, transpiration, and stomatal conductance were measured by an infrared gas analyzer, while the leaf water potential of the different leaves was determined by a digital pressure chamber. Canopy CO_2_ fluxes were measured hourly by closed canopy chamber^38^ for different growing stages. Also, canopy temperature was quantified for the whole growing season using an infrared radiometer sensor. Plant transpiration was automatically measured by sap flow sensors based on the heat balance method^39^. Data on crop growth processes (phenology, plant height, biomass, leaf area index, and yield) were collected every two weeks to capture dynamics of crop responses to soil and water conditions. Atmospheric weather conditions were also recorded at high temporal resolution. Associated agronomic management practices for each growing season (sowing density and depth, fertilization, pest treatments, etc.) are completely recorded.

## Methods

### Study locations and field design

The study area was located within the TERENO (TERrestrial ENvironmental Observatories) Eifel/Lower Rhine observatory near Selhausen in North Rhine-Westphalia, Germany (50°52^′^ N, 6°27^′^ E). Two minirhizotron facilities were set up in the field: the minirhizotron facility on the upper terrace with stony soil (hereafter R_UT_) contains up to 60% gravel by weight, while in the minirhizotron facility at the lower terrace with silty soil (hereafter R_LT_) the gravel content was approximately 4%. Soil types of R_UT_ and R_LT_ are Cambisol and stagnic Luvisol (FAO soil taxonomy - IUSS Working Group WRB^40^), respectively which are typical in the cropping fields in Rur catchment^41^.

**Fig. 1:**
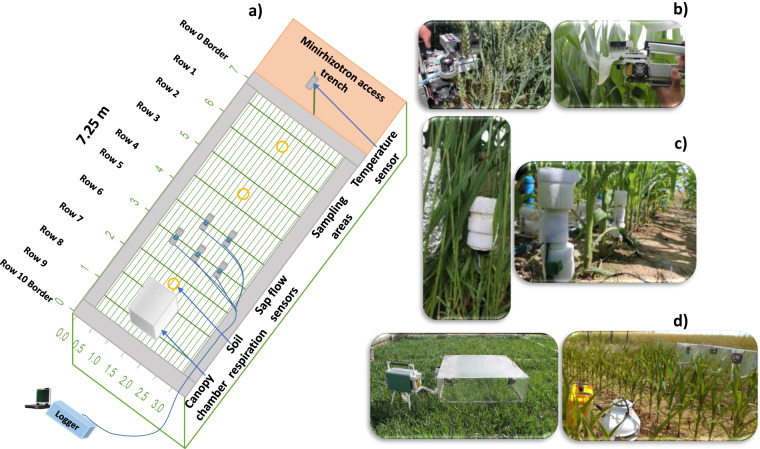
Overview of (**a**) above-ground measurement set-up in one exemplary plot (here for maize) within the minirhizotron facility, from the minirhizotron access trench: canopy temperature sensor, sampling area and leaf measurements, sap flow sensor installation with data logger, canopy chamber with LI-6400XT, and soil respiration chambers) (**b**) leaf gas exchange measurement with LI-6400XT machines (**c**) sap flow measurements with the Dynamax sensors, and (**d**) canopy chamber measurements for winter wheat (left) and maize (right).

The experimental site was divided into three plots (Plot 1, Plot 2, and Plot 3). The dimensions of each plot were 7.25 m × 3.25 m (Fig. 1). The experiment was performed from 2016 to 2021 and focused on crop responses (same cultivars of wheat and maize) to different soil types and water treatments (sheltered, rainfed, and irrigation). More specifically, winter wheat was tested in 2016 and 2021, while maize was tested in 2017, 2018, and 2020. The experiment was performed from 2016 to 2018 which focused on crop responses of wheat and maize with different stomatal behaviors to different soil types and water treatments (sheltered, rainfed, and irrigation). Wheat and maize cultivars were selected from the cultivars that were commonly grown in the farmers’ field. The experiment from 2020 and 2021 aimed to investigate the effects of cultivar mix and dry and wet conditions on cultivar performance. Two cultivars of each species with similar phenological states and maturity time were selected. Also, one cultivar was high performing in wet/normal years and low performing in dry years whereas the other was performing moderately under all conditions. We refer the readers to the twin paper^37^ for the detailed experimental set-up, where we focus here on describing the acquired methods and availability of above-ground data and agronomic practices (Table 1).

**Table 1:** Overview of data collections during growing seasons 2016, 2017, 2018, 2020, and 2021.

Variables, growing seasons, and crop species	2016 Winter wheat	2017 Maize	2018 Maize	2020 Maize	2021 Winter wheat
**Gas fluxes (hourly)**					
Leaf stomatal conductance	x	x	x	x	—
Leaf photosynthesis	x	x	x	—	—
Leaf water potential	x	x	x	—	—
Canopy gas chamber	x	x	x	—	—
Sap flow	x	x	x	x	—
Soil respiration	—	x	x	—	—
**Crop growth (bi-weekly)**					
Phenology	x	x	x	x	x
Stem/tiller number	x	x	x	x	x
Height	x	x	x	x	x
Aboveground dry matter (different organs)	x	x	x	x	x
Green leaf area index	x	x	x	x	x
Leaf greenness (SPAD values)	x	x	x	—	—
Plant C:N content	x	x	x	—	—
**Canopy temperature (hourly)**	x	x	x	—	—

### Leaf gas exchange and leaf water potential

Leaf gas exchange was measured biweekly in sunny days using a LI-6400XT Portable Photosynthesis System (Li-Cor Biosciences) with a given CO_2_ concentration of 400 ppm, constant flow rate of 500 (μmol s^−1^), and under ambient weather conditions (Table 2). For winter wheat in 2016, hourly leaf stomatal conductance, net photosynthesis, and transpiration of three to four of the fully developed leaves (top canopy) were measured at steady-state. In maize, the sunlit leaf are more important than the shaded leaf due its larger greenness area for intercepted radiation and assimilation. Since plot size and number plants were limited for the destructive sampling, in 2017 and 2018, two maize sunlit leaves (top canopy) and one shaded leaf were measured. Climatic variables from the leaf chamber like photosynthetic active radiation (PAR) (real time values from 27 to 2041 µM m^−2^ s^−1^), vapor pressure deficit (VPD), and leaf temperature were also recorded by the instrument. After the measurements of leaf stomatal conductance and photosynthesis, leaf were quickly cut by a sharp knife and inserted into a digital pressure chamber [(SKPM 140/ (40-50-80), Skye Instrument Ltd, UK] with the air pressure (0 to 35 bars) for leaf water potential (LWP) measurement. To investigate the full diurnal course of LWP and effects of irrigation, two days before and one day after irrigation with predawn measurements were carried out in 2018. In 2020, a leaf porometer (SC-1, Decagon Devices, Pullman, Washington, USA) (Table 1) was used to measure vapor pressure and humidity, and leaf stomata conductance. These measurements were done between 11 AM and 12 PM in the R_UT_ and R_LT_, respectively on four leaves of different plants per plot (and per cultivar in Plot 2) (Table 2).

**Table 2:** Detailed overview dates and time duration with measurement of leaf stomatal conductance, photosynthesis, and leaf water potential in the growing seasons 2016, 2017, 2018 and 2020 (date format dd.mm.yyyy).

No.	2016		2017		2018		2020	
1	20.04.2016	11:00-19:00	05.07.2017	9:20-18:00	15.06.2018	9:15-16:25	12.06.2020	11:00-12:00
2	06.05.2016	8:00-20:00	17.07.2017	13:40-18:30	20.06.2018	11:10-18:00	19.06.2020	11:00-12:00
3	25.05.2016	8:15-19:00	18.07.2017	8:00-19:00	27.06.2018	9:25-18:00	26.06.2020	11:00-12:00
4	26.05.2016	8:00-20:00	02.08.2017	9:10-17:00	03.07.2018	7:40-18:20	02.07.2020	11:00-12:00
	07.06.2016	8:30-14:00	04.08.2017	8:30-19:00	05.08.2018	8:30-17:45	14.07.2020	11:00-12:00
6	09.06.2016	7:00-20:00	07.08.2017	13:45-19:00	08.07.2018	7:30-19:30	28.07.2020	11:00-12:00
7	20.06.2016	7:40-14:20	13.08.2017	9:00-17:30	09.07.2018	11:00-17:30		
8	23.06.2016	7:20-20:15	16.08.2017	9:30-18:00	10.07.2018	8:30-17:00		
9	29.06.2016	7:30-20:30	23.08.2017	8:45-17:30	17.07.2018^*^	8:20-18:30		
10	08.07.2016	7:30-20:00	05.09.2017	9:10-16:45	18.07.2018^*^	7:00-19:40		
11	—	—	—	—	19.07.2018^*^	8:00-18:00		
12	—	—	—	—	26.07.2018	7:30-17:00		
13	—	—	—	—	02.08.2018	8:00-15:00		
14	—	—	—	—	16.08.2018	8:15-18:30		

Notes: ^*^ indicates the dates with leaf water potential was measured at predawn (4 AM).

### Sap flow

Sap flow measurement followed the stem heat balance basics (Eq. 1) where a given power input to stem (Pin, W) is equal to the sum of the vertical or axial heat conduction through the stem (Q_v,_ W), radical heat conducted through the sensor gate to the ambient (Q_r,_ W), and the heat convection varied by the sap (Q_f_, W) (Dynamax, 2005)^42^.

1 $${Pin}={Q}_{v}+{Q}_{r}+{Q}_{f}$$

Sap flow rate per unit of time (F, g h^−1^) is calculated by dividing the residual of energy balance to the temperature increase of the sap (dT, °C) and the heat capacity of water (C_p_, joules g^−1^ °C^−1^) in Eq. (2).

2 $$F=\frac{\,{Q}_{f}}{{C}_{p}\ast {dT}}\ast 3600$$

**Fig. 2:**
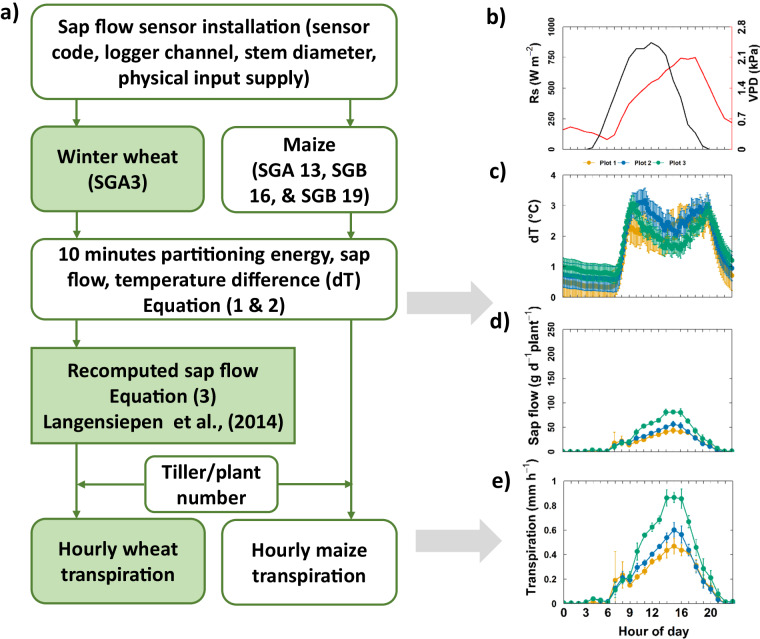
Sap flow measurement (**a**) routines to acquire and process the sap flow data for winter wheat and maize [SGA and SGB are types of sap flow sensor, dT (°C) is the temperature differences between upper and lower thermocouples in the sensor] and an example of diurnal (**b**) global radiation (Rs, W m^−2^) and vapor pressure deficit (VPD, kPa) (**c**) temperature differences (dT, °C), (**d**) average sap flow of single plant (g d^−1^ plant^−1^), and (**e**) canopy transpiration (mm h^−1^) from three plots (Plot 1, 2, and 3) at the upper minirhizotrone (R_UT_) on 21^st^ July, 2017.

In 2016, sap flow sensors were installed when wheat stem diameter was between 3–5 mm (26 May). Five, three, and five sensors (SAG3) (Dynamax Inc., Houston, USA) in the irrigated, rainfed, and water-stressed plots, respectively were operated until harvest. The energy partitioning, temperature difference of thermocouples (dT), power supply, and calculated sap flow of each sensor were recorded every ten minutes using a CR1000 data logger and two AM 16/32 multiplexers (Campbell Scientific, Logan, Utah). Because of the thin and hollow stem of wheat, the majority of heat energy input was diverted to radial heat flow, leaving only little energy (Q_f_) partitioned to convective heat flow^39^. The sap flow was re-estimated based on the empirical function with k factor (g °C^−1^ h^−1^) and dT (°C) alone (Eq. 3 and Fig. 2a) following the approach of Langensiepen *et al*.^39^. We used a constant value for k (k = 0.45 g °C^−1^ h^−1^) for all sensors and the whole measured period.

3 $$F=k\ast {dT}$$

Sap flow was processed separately for each sensor and each minirhizotrone. The 10-minute data (dT °C) from the raw data were aggregated to hourly values. The hourly dT value of each sensor for each day was subtracted to the minimum value of that day which allows to offset values of dT before estimating sap flow by Eq. (3). Sap flow of in single tillers was upscaled to canopy transpiration based on the bi-weekly recorded tiller number. For the post-processing step, the raw input signal file (e.g. “RUT_2016_Sap_Flow_Raw.csv”, and “RUT_2016_Tiller_Number.csv”), the R scripts (e.g. “RUT_2016_Sap_Flow_Code_Process.R”), and intermediate and final sap flow data output (e.g. “RUT_2016_Sap_Flow.csv”) were provided in the repository folders.

In 2017 (from 8^th^ July 2017 until 11^th^ September 2017) and 2018 (from 29^th^ June 2018 and 06^th^ July for R_UT_ and R_LT_, respectively until 21^st^ August 2018), 15 sap flow sensors (SGA 13, SGB 16, and SGB 19 types) were installed on 5 maize plants per plot based on stem diameter size. Likewise, in 2020 (from 31^st^ July 2020 until 01^st^ September 2020), 32 sap flow sensors (SGA 13, SGB 16, and SGB 19 types) were installed (16 in the R_UT_ and 16 in the R_LT_) (Table 3). Sensors were placed on 4 maize plants within Plot 1, 4 plants within Plot 3, and 8 plants in the varietal mixture Plot 2 (4 per variety). Due to COVID-19 restrictions, sap flow measurement was not performed in 2021. Unlike winter wheat, the energy (Q_f_) partitioned to convective heat flow in maize stem was sufficient (e.g. Q_f  _ > 20% of P_in_) that the sap flow of maize plant (g h^−1^) was estimated directly based on the Eqs. (1 & 2) by the data loggers (Dynamax, 2005)^42^ (Fig. 2a). The post-process R scripts were employed to perform quality check for the raw sap flow data of each sensor and each minirhizotrone facility. The number of plants per square meter was multiplied with the average sap flow of single plants to estimate canopy transpiration (Fig. 2b–d).

**Table 3:** Detailed overview of number of sensors, dates, and duration with measurement of sap flow in the growing seasons 2016, 2017, 2018, 2020, and 2021 with the number of sensors in each plot (date format dd.mm.yyyy).

Rhizotrone facility	Plot	2016		2017		2018		2020		2021
R_UT_	1	5	26.05.2016-26.07.2016	5	08.07.2017-11.09.2017	5	29.06.2018-21.08.2018	4	29.06.2020-21.08.2020	—
R_UT_	2	3	26.05.2016-26.07.2016	5	08.07.2017-11.09.2017	5	29.06.2018-21.08.2018	8	29.06.2020-21.08.2020	—
R_UT_	3	5	26.05.2016-26.07.2016	5	08.07.2017-11.09.2017	5	29.06.2018-21.08.2018	4	29.06.2020-21.08.2020	—
R_LT_	1	5	26.05.2016-26.07.2016	5	08.07.2017-11.09.2017	5	06.07.2018-21.08.2018	4	06.07.2020-21.08.2020	—
R_LT_	2	3	26.05.2016-26.07.2016	5	08.07.2017-11.09.2017	5	06.07.2018-21.08.2018	8	06.07.2020-21.08.2020	—
R_LT_	3	5	26.05.2016-26.07.2016	5	08.07.2017-11.09.2017	5	06.07.2018-21.08.2018	4	06.07.2020-21.08.2020	—

### Canopy chamber measurement

**Fig. 3:**
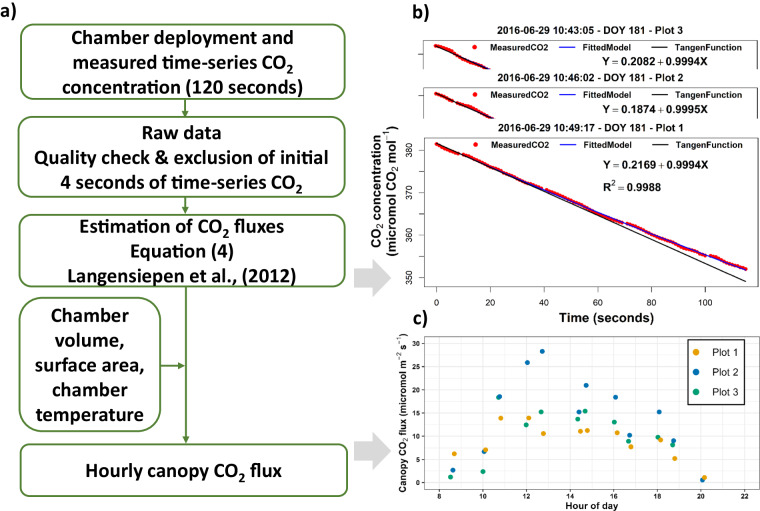
Canopy CO_2_ gas flux based on the closed canopy chamber (**a**) overview of data collection and processing and an example of (**b**) the computation of CO_2_ flux based on the CO_2_ time-series, and (**c**) a diurnal course of hourly canopy CO_2_ flux in three plots (Plot 1, 2, and 3) in the R_UT_ on 29^th^ June, 2016.

Canopy gas exchange was measured hourly on mostly same days with the leaf gas exchange and leaf water potential measurements (Table 4). A closed and transparent plexiglas-chamber system (Langensiepen *et al*.)^38^ was used to determine the time series of CO_2_ change using an infrared gas analyzer (LI-6400XT Portable Photosynthesis System(Li-Cor Biosciences, Lincoln, Nebraska, USA) (Fig. 1d). One chamber was put in each plot. The chamber width and length were 100 cm × 100 cm with a height that could vary to adapt to the crop height. A time series of CO_2_ concentration (120 seconds) together with relevant inputs (inside chamber temperature, humidity, and PAR were recorded. Due to the perturbation of environment when deploying the chamber, the first 4 seconds of each CO_2_ time series were excluded (Fig. 3). The saturation regression approach^38^ was employed to estimate fluxes rates. The CO_2_ flux was estimated by Eq. (4) where *F*_*CO2*_ is flux of CO_2_ (micromole m^−2^ s^−1^), *p* is the air density mole m^−3^, *V* is the chamber volume (m^3^), *S* is the ground surface area (m^−2^), and *dC/dt* is the concentration changes over time (mole s^−1^).

4 $${F}_{{CO}2}=\frac{p\ast V}{S}\ast \frac{{dC}}{{dt}}\ast {10}^{6}$$

**Table 4:** Detailed overview dates and duration with measurement of canopy CO_2_ flux in the growing seasons 2016, 2017 and 2018 (date format dd.mm.yyyy with local time).

Number	2016		2017		2018		2020	2021
1	07.04.2016	11.00-12.00	16.06.2017	8.30-17.00	15.06.2018	8:45-17:00	—	—
2	11.04.2016	10.00-17.00	21.06.2017	8.00-18.00	20.06.2018	10:00-18:00	—	—
3	20.04.2016	9.00-16.00	05.07.2017	8.30-18.00	27.06.2018	9:00-17:00	—	—
4	06.05.2016	8.00-19.00	17.07.2017	14.00-19.00	05.07.2018	10:30-17:00	—	—
5	26.05.2016	9.00-16.00	18.07.2017	8.30-18.00	17.07.2018	10:00-17:00	—	—
6	09.06.2016	10.00-19.00	31.07.2017	11.30-18.00	19.07.2018	9:30-17:00	—	—
7	20.06.2016	9.00-14.00	02.08.2017	9.00-17.00	02.08.2018	10:00-17:00	—	—
8	29.06.2016	8.30-20.00	16.08.2017	10.00-17.00	16.08.2018	10:00-16:00	—	—
9	15.07.2016	9.00-17.00	29.08.2017	10.00-18.00	—	—	—	—

For the post-processing step, the raw input signal file (e.g. “RUT_2016_Canopy_CO2_Flux_Raw.csv), the R scripts (e.g. “RUT_2016_Canopy_CO2_Flux_Code_Process.R”), and intermediate and final CO_2_ gas flux data output (e.g. “RUT_2016_Canopy_CO2_Flux.csv”) were provided in the repository folders.

### Soil respiration

Hourly respiration was manually measured by a portable soil CO2 flux system (LI-8100, Li-Cor Biosciences) (Fig. 1a,d) which was mostly in parallel with the canopy chamber measurements in 2017 and 2018 (Table 5). It measured the CO_2_ efflux from the soil which includes the respiration from plant roots and microorganisms surrounding the roots, and from heterotrophic microorganisms that metabolize plant litter and soil organic matter. The CO_2_ concentration was recorded in 90 seconds on the area of 317 cm^2^ and a volume of 5319.6 cm^3^ placed on a plastic ring with an inner diameter of 20.09 cm. Two and three rings were installed in each plot in 2017 and 2018, respectively (see also soil respiration in Fig. 1). The calculation of CO_2_ flux was done based on the exponential fit of the CO_2_ time series using the LICOR application LI-8100 file viewer.

**Table 5:** Detailed overview dates and duration with measurement of soil respiration flux in the growing seasons 2016, 2017 and 2018(date format dd.mm.yyyy with local time).

Number	2016	2017		2018		2020	2021
1	—	16.06.2017	—	15.06.2018	—	—	—
2	—	21.06.2017	—	20.06.2018	10:20-18:15	—	—
3	—	05.07.2017	8:30-19:00	27.06.2018	9:20-16:55	—	—
4	—	17.07.2017	—	05.07.2018	—	—	—
5	—	18.07.2017	12:30-17:00	17.07.2018	7:50-16:00	—	—
6	—	31.07.2017	11:30-18:15	19.07.2018	7:30-17:00	—	—
7	—	02.08.2017	—	02.08.2018	—	—	—
8	—	16.08.2017	9:40-17:30	16.08.2018	—	—	—
9	—	23.08.2017	9:45-17:50	—	—		
10	—	29.08.2017	10:00-18:00	—	—	—	—
11	—	05.09.2017	10:50-16:30	—	—		

### Crop growth measurement

The crop phenology was observed based on BBCH-scale (Biologische Bundesanstalt, Bundessortenamt und CHemische Industrie). The crop growth data (total aboveground dry matter, green and brown leaf area, dry matter of different organs, leaf greenness, wheat tiller number, plant height, and stem diameter) was determined bi-weekly (see the data summary in Table 1 and Table 6). The total aboveground dry matter and leaf area were determined from 10, 7, and 10 dates for 2016, 2017, and 2018, respectively. Because of the limited plant number in each plot, we sampled two rows (one meter each) in winter wheat in 2016 and two plants in maize in 2017 and 2018 (Table 6). For 2020, three maize plants per plot (and per variety in Plot 2) were taken for each sampling date (in total 6 dates). In 2021, two rows (one meter each) at three sampling points per plot were sampled (in total 4 dates). At the final harvest, in each plot, five replicates (three replicates with one meter square each and two replicates with one row each) were sampled for winter wheat to determine dry matter and grain yield in 2016. In 2017 and 2018, five separate replicates (one meter square each) were collected for maize. Nine rows (two meters each) of maize and six rows (one meter each) of wheat per Plot (and per variety in Plot 2) were sampled in 2020 and 2021, respectively. Fresh biomass samples were first weighed in total. Different plant organs (green leaf, brown leaf, stem, ear, and grain) were then separated and weighed. Green and brown leaf area were measured in the lab using leaf area meter (LI-3100C, Li-Cor Biosciences). Subsamples were taken afterward from these organ samples, weighed, then dried for 48 h in an oven at 105 °C. Dry subsamples were weighed again for determining dry matter. Another part of each plant organ, e.g., green leaves, brown leaves, stems, and ears or grains was analyzed for nitrogen and carbon content. The samples were oven dried at 60 °C for at least 24 hours, then milled and weighed. Repeat determinations (four times) were conducted for each plant organ with a Euro Elemental Analyzer (Euro EA-CHNSO Elementanalysator, HekaTech GmbH). Leaf greenness was measured from 12 random leaves using a SPAD Chlorophyll-meter (Konica Milta, Inc., Japan). Plant height was determined for five random plants in case of winter wheat (2016 and 2021). In 2017 and 2018, the plant height was measured from 15 plants, while 5 plants were measured in 2020. Stem diameter was measured randomly from five main tillers (wheat) and stems (maize) around the sap flow installation. In addition to the above-ground data, root data was also measured by root coring which is described in the twin paper^37^.

**Table 6:** Detailed overview of dynamic crop growth information (leaf area, biomass, and grain yield) during growing seasons 2016, 2017, 2018, 2020, and 2021 (date format dd.mm.yyyy).

Number	2016	2017	2018	2020	2021
1	20.04.2016	15.06.2017	15.06.2018	19.06.2020	13.04.2021
2	06.05.2016	29.06.2017	20.06.2018	02.07.2020	17.05.2021
3	25.06.2016	14.07.2017	27.06.2018	14.07.2020	08.07.2021
4	02.06.2016	27.07.2017	05.07.2018	28.07.2020	12.08.2021
5	09.06.2016	04.08.2017	11.07.2018	02.09.2020	—
6	15.06.2016	13.08.2017	19.07.2018	23.09.2020	—
7	20.06.2016	23.08.2017	26.07.2018	—	—
8	23.06.2016	12.09.2017	02.08.2018	—	—
9	29.06.2016	—	16.08.2018	—	—
10	08.07.2016	—	22.08.2018^*^	—	—
11	26.07.2016	—	30.08.2018^¥^	—	—

Notes: ^*^ indicates harvest date for plot 2, 3 (R_UT_) and plot 1, 2, 3 (R_LT_) while ^¥^ indicates harvest date for plot 1 (R_UT_) in 2018.

### Canopy temperature

The canopy temperature was measured every 30 minutes by an infrared radiometer sensor (model Apogee SI-121, UP Umweltanalytische Produkte GmhH) and C1000 data logger (Campbell Scientific, Utah, USA). Sensor (one each plot) was installed at 2 and 2.5 m above the soil surface with a measured angle of 45° for winter wheat and maize, respectively Fig. 3.

## Data Records

**Fig. 4:**
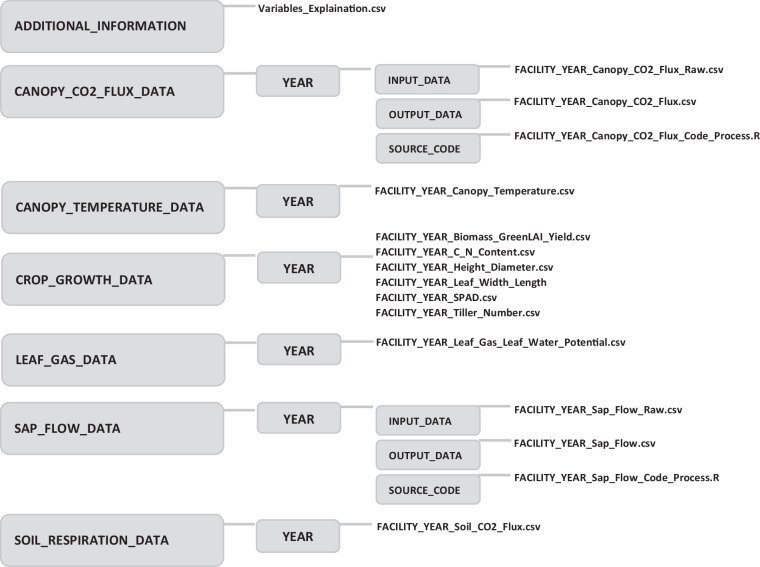
Names and structure of the data folder in the repository.

All data were uploaded to Geonetwork in accordance with ISO 19115. The data were permanently stored and will be regularly updated (see Usage Notes). The above-ground data was organized based on data types and measurements into separate folders (Fig. 4). The “CROP_GROWTH_DATA” folder contains crop growth parameters [dynamic biomass, green leaf area index, crop height, stem diameter, tiller number (for wheat), C: N content, SPAD values, and final yield]. Hourly leaf gas exchange (photosynthesis, stomatal conductance, transpiration) and leaf water potential were put into the “LEAF_GAS_DATA” folder. The hourly sap flow data was located at the “SAP_FLOW_DATA” folder. “CANOPY_CO2_FLUX_DATA”, “SOIL_RESPIRASION_DATA”, and “CANOPY_TEMPERATURE_DATA” were devoted for the hourly canopy CO_2_ flux data, soil respiration, and canopy temperature, respectively (Fig. 4). Each of those folders contains different “YEAR” folders. Each folder contains two “.csv” files corresponding to the data of two minirhizotrone facilities (R_UT_ and R_LT_). Names, explanation, and units of the measured variables were described in the “ADDITIONAL_INFORMATION” folder with the file “Variables_Explaination.csv”. For the sap flow and canopy CO_2_ flux data, three additional folders were created. The “INPUT_DATA” folder contains all raw input data, while the “SOURCE_CODE” folder contains the R scripts with detailed steps to process raw data for each minirhizotrone facility. The “OUTPUT_DATA” folder contains the intermediate files (e.g. graphs or final data in the “.csv” file). All data, source codes, intermediate variables (e.g. sap flow and CO_2_ fluxes), and additional information were stored in one folder are provided in the repository^43^.

## Technical Validation

### Leaf and canopy gas exchange data by two LI-6400XTs

The two LI-6400XT and soil respiration chamber LI-8100 were calibrated before every growing season to avoid gas leaking from the chamber head and cuvette during the measurement period. The calibration was performed with two sequent steps: zeroing with compressed gas and setting the CO_2_ span with “IRGA (infrared gas analyzer) Zero” and “IRGA Span”, respectively (e.g. in LI-6400XT manual version 6.2, page 18-10^44^). The former step was carried out via directly connecting the sensor head with the compressed CO_2_ free air (room temperature, bypass the console, and pump-off) using “Y” connector and moderate flow rate (c.a. 500 ml min^−1^). After the first step, the span of CO_2_ analyzer (using 418 ppm of CO_2_ from the compressed gas) and H_2_O analyzer (using water concentration from LI-610 Dew point generator) were performed at room and dew point (15 °C), respectively. Temperature sensors inside and outside the chamber/cuvette, vapor pressure deficit sensor, and photosynthetically active radiation sensors were also checked to give reasonable values before deploying. The compartments of the transparent Plexiglas canopy chambers were also examined for gas leakages. The CO_2_ and H_2_O concentration curves were displayed in the console during operation. Abnormal curves (e.g. sudden changes of values) were discarded and replaced by a subsequent measurement. Due to the environmental disturbances when deploying the canopy chambers, initial values (4 seconds) of the CO_2_ time-series were removed before calculating CO_2_ flux.

### Leaf stomata conductance data by porometer

Two humidity sensors in the head of the leaf porometer must act in a fairly predictable way to produce reliable readings of stomatal conductance. Before taking measurements on each sample day, the porometer was calibrated through the procedure indicated in the manual (SC-1 Leaf porometer manual version 1.07).

### Sap flow data

Sap flow sensors were manually investigated to detect sensor malfunction (Dynamax manual, 2007). For instance, socket connections and/or Ohm values of the sensors were checked and recorded for each growing season. The sensors with either loss of connection or with resistivity values that were considerably different from the default values were not used. Cable functions were also checked to ensure proper connections between sensors and the data loggers. During an operation of the sensor in the field, the plausibility of sap flow signals (e.g. energy partitioning and dT) was checked regularly using the Dynamax software installed in a tough book.

### Leaf area index data

The transparent plastic band in the leaf area meter LI-3100C was carefully cleaned before use and regularly checked to avoid unintended dirt that could influence the measurements of leaf area.

### Shoot and grain C: N content data

The Euro Elemental Analyzer needs to be calibrated with subsamples in each round of analyzing. Implausible values of C and N were manually determined from the calibration.

## Usage Notes

### Data availability

Tables 1, 2, 3, 4, 5, 6, and Fig. 4 provide information on data availability across the growing season and different data variables. Data was collected and managed by two different projects funded by German Research Foundation (DFG). Data from 2016-2017-2018 belonged to the project B5-SFB-TR32-Transregional collaborative research program, while the data from 2020–2021 has belonged to the project EXC-2070-375 390732324 – PhenoRob. Crops were not sown on the minirhizotron facilities in 2019 due to project change and maintenance works of the rhizotrone cellars. The management practice information was recorded from sowing to harvest, while crop growth and flux data were measured for certain days during the main growing season (Tables 2, 3, 4, 5, and 6). Crop growth data (biomass, height, leaf area index, and final harvest) (Table 6) were performed every year (2016, 2017, 2018, 2020, and 2021). Sap flow was not measured in 2021 because of COVID-19 restrictions (Table 3). Soil respiration was not measured in 2016, 2020, and 2021. Leaf photosynthesis, leaf water potential, canopy CO_2_ flux (with the closed chamber), and canopy temperature were not measured in 2020 and 2021.

### Updates

The above-ground crop growth and sap flow data which were collected in 2022 and in coming years will be updated after data analysis and quality control. The data will be uploaded to the same data repositories as in Fig. 4.

### Below-ground Data

The related below-ground data (e.g. soil characteristics, soil water dynamics, and root data) was collected and managed by the Institute of Bio and Geoscience, Agrophere (IBG-3) of Jülich Research Center which is published in a twin paper^37^. A part of the below-ground data of wheat (2016) and maize (2017) (root length density obtained from manual single root annotation, soil water content, and soil texture) has been published in Nguyen *et al*.^19^ and Nguyen *et al*.^18^, respectively. Root length data obtained from the images and the soil moisture measured by TDR and MPS-2 sensors on both facilities in 2016 and 2017 were used in Morandage *et al*.^45^. The root images and root length data of the R_UT_ and R_LT_ in 2017 and 2018 were published in Bauer *et al*.^46^ and Nguyen *et al*., (under review)^47^. The GPR data and the mean soil water content which were calculated from the TDR sensors in 2016 and 2017 have been partly used and published in Klotzsche *et al*.^48^ and Lärm *et al*.^49^. The destructively sampled root data by soil cores (2020 and 2021) is available in the twin paper from Lärm *et al*.^37^.

## Data Availability

Custom codes were used to process the raw data (e.g. for sap flow and canopy CO_2_ fluxes). These data were processes by codes writing in R (version 4.3.1). Custom codes for sap flow processes and CO_2_ fluxes are provided in the data repository.
